# The value of a non-invasive bladder sensitivity paradigm in chronic pelvic pain

**DOI:** 10.1530/RAF-25-0091

**Published:** 2026-03-04

**Authors:** Lydia Coxon, Emily Tan, Michal Krassowski, Pedro Abreu Mendes, Joana Ferreira Gomes, Claire E Lunde, Jane Meijlink, Danielle Perro, Lars Arendt-Nielsen, Qasim Aziz, Christian M Becker, Judy Birch, Ana Charrua, Lysia Demetriou, Emma Evans, Anja Hoffman, Andrew W Horne, Lone Hummelshoj, Stacey A Missmer, Eloise Passarella, Esther Pogatzki-Zahn, Rolf-Detlef Treede, Jan Vollert, Krina T Zondervan, Christine B Sieberg, Francisco Cruz, Jens Nagel, Katy Vincent

**Affiliations:** ^1^ University of Oxford, Oxford, UK; ^2^ IBMC and Faculty of Medicine of University of Porto, Porto, Portugal; ^3^ Boston’s Children’s Hospital, Boston, USA; ^4^ International Painful Bladder Foundation, Naarden, The Netherlands; ^5^ Aalborg University, Aalborg, Denmark; ^6^ Department of Gastroenterology & Hepatology, Mech-Sense, Clinical Institute, Aalborg University Hospital, Aalborg, Denmark; ^7^ Steno Diabetes Center North Denmark, Clinical Institute, Aalborg University Hospital, Aalborg, Denmark; ^8^ Wingate Institute of Neurogastroenterology, Blizard Institute, Barts and The London School of Medicine and Dentistry, Queen Mary University of London, London, UK; ^9^ Pelvic Pain Support Network, Poole, United Kingdom; ^10^ Bayer AG, Research & Development, Pharmaceuticals, Berlin, Germany; ^11^ Centre for Reproductive Health, Institute of Regeneration and Repair, University of Edinburgh, Edinburgh, UK; ^12^ Endometriosis.org, London, United Kingdom; ^13^ Michigan State University, East Lansing, Michigan, USA; ^14^ University of Calgary, Calgary, Canada; ^15^ University Hospital Muenster, Muenster, Germany; ^16^ Heidelberg University, Mannheim, Germany; ^17^ Department of Clinical and Biomedical Sciences, Faculty of Health and Life Sciences, University of Exeter, Exeter, UK; ^18^ Exploratory Pathobiology, Research & Development, Pharmaceuticals, Bayer Aktiengesellschaft, Wuppertal, Germany

**Keywords:** chronic pelvic pain, IC/BPS, bladder pain, endometriosis, visceral sensitivity

## Abstract

**Abstract:**

We explore bladder sensitivity profiles in females with chronic pelvic pain, using a non-invasive bladder sensitivity paradigm. We aim to determine how profiles differ between groups defined by symptoms; groups defined by diagnoses; and understanding how bladder sensitivity relates to clinical symptoms. A previously developed non-invasive bladder sensitivity paradigm will be used, which assesses pain and urgency ratings during physiological bladder filling, time to reach descriptive sensations, and volume voided at the paradigm end. Our cohort comprised individuals with (pain + bladder) and without (pain only) overt bladder symptoms and pain-free/bladder-symptom-free controls; within both pain groups were participants with and without endometriosis. In addition, questionnaires were completed, which included bladder- and bowel-related symptoms. Participants with pain + bladder symptoms had significantly greater pain intensity scores throughout the paradigm compared with controls. When looking at underlying diagnoses, the only significant difference between those with bladder symptoms, with and without endometriosis, was in the volume voided at the end of the paradigm. We found correlations between pain ratings during the paradigm and gastrointestinal symptom scores from questionnaires. We found that the paradigm can distinguish between those with overt bladder symptoms and controls. It also appears to potentially identify a group of individuals with ‘silent’ bladder symptoms. It is currently unclear whether those with ‘silent’ symptoms go on to develop overt symptoms. Overall, our study highlights the need to explore bladder sensitivity in individuals with any form of chronic pelvic pain, as comorbidity is common and ‘silent’ symptoms are not well understood.

**Lay summary:**

This study explored bladder sensitivity in people with chronic pelvic pain, with and without bladder symptoms, compared with a control group of people without pain. We wanted to explore pain and urgency when people drank water and how this differed in people with different symptoms and diagnoses. We asked people to drink 20 fl oz of water and give ratings of pain and urgency throughout, as well as when they felt certain sensations. We found differences between those with overt bladder symptoms and the control group, with those with overt bladder symptoms experiencing greater pain. We did not find differences between different diagnoses (e.g. those with and without endometriosis) in pain or urgency ratings. This study highlights the need to explore bladder sensitivity in individuals with any form of chronic pelvic pain.

**Graphical Abstract:**

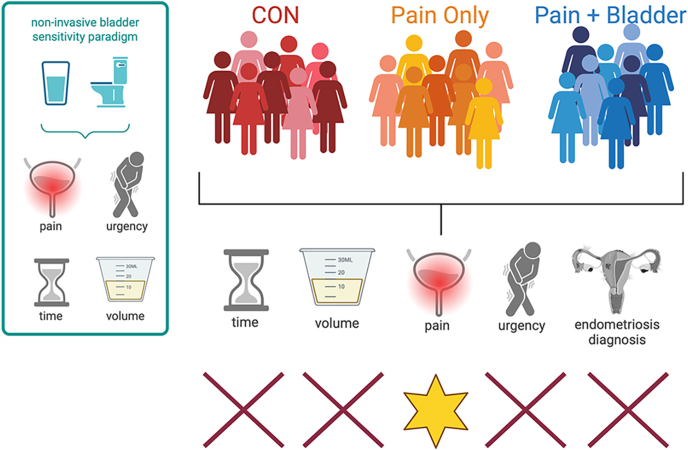

## Introduction

Chronic pelvic pain (CPP) affects up to 26.6% of women worldwide (Eskenazi & Warner 1997, Zondervan *et al.* 2001, 2018, Ahangari 2014) and is associated with a variety of symptoms and pathologies. Endometriosis and interstitial cystitis/bladder pain syndrome (IC/BPS) are two conditions in which there is a high prevalence of CPP (Dydyk & Gupta 2025). Whilst research has tried to better understand both these pathologies and other CPP conditions, our current understanding of CPP remains limited. The Translational Research in Pelvic Pain (TRiPP) study aims to address this knowledge gap, focussing on mechanisms giving rise to and maintaining pain, and if/how these relate to any underlying pathology/diagnostic label.

Bladder hypersensitivity is likely to be an important mechanism in CPP but has until recently been difficult to study, in part due to invasive testing methods (Kelly & Krane 2000, Andresen 2009). Tu *et al.* published a non-invasive bladder sensitivity paradigm (Tu *et al.* 2013), which enables testing of bladder sensitivity by inducing natural diuresis. Data produced from this paradigm are the times to different sensations, pain and urgency ratings (0–10) throughout, and volume voided at the end. This paradigm has been used in different populations of females with/without pelvic pain: adults with dysmenorrhoea, who were shown to have greater sensitivity than controls despite no overt bladder symptoms (Hellman *et al.* 2018, 2020, Oladosu *et al.* 2019); premenarchal adolescents, who demonstrated no significant difference between those who go on to have dysmenorrhoea and those that do not (Tu *et al.* 2024); and reproductive-aged-women, as part of a multimodal hypersensitivity model predicting pelvic pain after 4 years (Kmiecik *et al.* 2023). These suggest that this paradigm may have utility in distinguishing between patient groups, specifically in terms of identifying those with and without bladder hypersensitivity.

We therefore used this paradigm, in combination with questionnaire measures, to explore bladder hypersensitivity in females with CPP with the aims of determining how bladder sensitivity profiles differ between i) groups defined by symptoms and ii) groups defined by diagnoses and iii) of understanding how bladder sensitivity relates to clinical symptoms.

The TRiPP cohort comprised those with pelvic pain with and without bladder symptoms (subgroups with endometriosis) and those with neither pelvic pain nor bladder symptoms. We hypothesised that participants with pelvic pain would have greater pain scores than pain-free/bladder-symptom-free controls throughout the paradigm (reflecting increased sensitivity to visceral nociception). Furthermore, these pain scores would be higher in participants with bladder symptoms than in those without. We also hypothesised that participants describing bladder symptoms would reach predefined sensations in a shorter time (reflecting greater responses to distension), with controls reaching the longest time. Finally, given the heterogeneity in symptoms resulting from the complexity of mechanisms underlying visceral sensation and function (Roth *et al.* 2011, Souza *et al.* 2011, Stratton *et al.* 2015, Coxon *et al.* 2023), we did not expect that the presence of endometriosis diagnosis would influence findings, in participants both with and without bladder symptoms.

## Methods

### Participant recruitment

All participants were recruited to the Translational Research in Pelvic Pain (TRiPP) study as per published protocol (Demetriou *et al.* 2022). Recruitment and data collection occurred at three sites: University of Oxford, UK (OX); Boston’s Children’s Hospital, USA (BCH); and Instituto de Biologia Molecular e Celular, Portugal (IBMC). Participants from OX and BCH were selected based on study criteria, from an existing database of participants from parent studies. Participants from IBMC were recruited through urology clinics.

### Ethics statement

All appropriate ethical approvals were secured before recruitment into TRiPP (ethics reference 19/YH/0030).

### Participants

We only included those assigned female at birth, but we did not ask about gender identity. Participants in this study were categorised into five groups: EAP (previously received a surgical diagnosis of endometriosis and pelvic pain >4/10), EABP (meet EAP criteria with additional pain experienced in the bladder and urinary frequency and/or urgency symptoms), BPS (pain perceived to arise from the bladder >4/10 and urinary symptoms, with no surgical diagnosis of endometriosis), PP (pelvic pain >4/10 without bladder pain or urinary symptoms and no surgical diagnosis of endometriosis), and CON (controls with no history of endometriosis, no pelvic pain (all scores <3/10), and no urinary symptoms) (see Supplementary Table 1 for details (see section on Supplementary materials given at the end of the article)). All our groups are based on clinical presentation (with the exception of surgical confirmation of endometriosis diagnosis) as people are frequently over- and under-diagnosed when the complexity of chronic pelvic pain is not fully understood. All participants were females aged 18–50 at recruitment and were neither pregnant nor lactating. All participants gave informed consent.

### Data collection

Participants completed questionnaires including the O'Leary-Sant Interstitial Cystitis Symptom and Problem Index (ICSI and ICPI) (O’Leary *et al.* 1997) and the Gastrointestinal Symptom Rating Scale (GSRS) (Svedlund *et al.* 1988), which are used here, and several other pain-relevant questions/validated scales (Demetriou *et al.* 2022). They were also asked about bladder pain intensity ratings at its worst and on average over the last 3 months using a numerical rating scale (NRS) (0–10). Bladder pain interference in the last 3 months was assessed using a Likert scale (not applicable, not at all, slightly, moderately, quite a bit, and extremely), broken into work or school, daily activities at home, sleep, sexual intercourse, exercise/sports, and social activities.

They were also asked to complete a questionnaire on the day of testing, which included current pain intensity (NRS 0–10), caffeine and medications taken in preceding 24 h, date of last menstrual cycle, and state anxiety (Spielberger & Lushere 1971).

As per the published paradigm (Tu *et al.* 2013), participants were asked to void so the paradigm starts with an empty bladder. Then, participants were given 20 fl oz (US) water to drink in 5 min and were asked for pain and urgency ratings (NRS 0–10 from no pain/urgency to worst pain/urgency imaginable) every 15 min. Time started when they started to drink. They were asked to state when they reached key sensations: first sensation (FS) (described as ‘when riding in a car, the driver pulls over to a rest-stop to urinate, you would go as well’), first urge (FU) (‘when riding in a car, you would initiate the request to find a rest-stop to urinate’), and maximum tolerance (MT) (‘when riding in a car, you would urinate on the side of the road in bumper-to-bumper traffic’) (Abrams *et al.* 2002). At each sensation the time, pain and urgency ratings (NRS) were recorded. If MT was not reached at 45 and 60 min, additional 10 fl oz water was given (maximum additional 20 fl oz). The participant voided at either MT or after 2 h (if MT not reached), and the volume of urine voided was measured.

Participants self-reported whether they were taking hormonal contraceptives, the day of their last menstrual period, and typical length of their menstrual cycle. Those who were not currently taking hormonal contraception, who indicated that they had menstrual cycles, were categorised by menstrual phase as in previous TRiPP manuscripts (Coxon *et al.* 2023), and this was cross-checked by two researchers (LC and DP).

### Statistical analysis

All data were double-entered into databases and analysed using SPSS 27 and GraphPad Prism 10.

Analysis was carried out between those in symptom groups, with pelvic pain and bladder symptoms (pain + bladder; EABP and BPS), those with pelvic pain without bladder symptoms (pain only; EAP and PP), and pain-free/bladder-symptom-free controls (CON). Then, analysis was carried out between diagnostic groups using TRiPP groups (EAP, EABP, PP, and BPS), excluding CON. This allowed greater power to try to distinguish whether the presence of bladder symptoms, rather than diagnosis per se, resulted in differences between groups.

Descriptives of median, interquartile range (IQR), and (range) were calculated. For between-group comparisons, Kruskal–Wallis, Mann–Whitney U, and Dunn’s tests were used, with 95% confidence intervals calculated using weighted average. For correlation analysis, Spearman’s correlation was used; this was only in participants who had pelvic pain (e.g. excluding the CON group). Bonferroni correction was applied for multiple comparisons.

Area under the curve (AUC) analysis was used for both pain and urgency over the experimental timeframe, with the proportional area reported. This was calculated using the actual AUC for an individual divided by the maximum AUC possible for that individual, depending on the total time of the paradigm. This was needed due to the varying length of the paradigm between individuals. Proportional area was then compared between groups.

## Results

### Participants and demographics

We recruited 71 participants – EAP: *n* = 18, EABP: *n* = 11, BPS: *n* = 14, PP: *n* = 5, and CON: *n* = 23. The demographics of the groups are shown in Table 1, visceral symptoms assessed by questionnaires in Table 2, and variables collected on the day of testing, including pain rating and menstrual cycle phase, in Table 3.

**Table 1:** Demographics of the diagnostic groups. Data are presented as median (IQR) and range or as *n* (%) as appropriate. For race, participants could select multiple options or leave the response blank.

	CON	Pain only		Pain + bladder	
		EAP	PP	EABP	BPS
Participants, *n*	23	18	5	11	14
Age, years					
Median (IQR)	30 (26–39)	33 (25.5–37)	33 (28–33.5)	34 (27–39)	47 (34.5–50.25)
Range	21–45	22–47	27–34	25–48	27–51
Parity					
Median (IQR)	0 (0–0)	0 (0–0)	0 (0–0)	0 (0–1)	1 (0–2)
Range	0–2	0–2	0–0	0–1	0–4
Race, *n* (%)					
Alaskan	1 (4.3)	0 (0)	0 (0)	0 (0)	0 (0)
Asian	3 (13.0)	0 (0)	0 (0)	1 (9.1)	0 (0)
Black	2 (8.7)	1 (5.6)	1 (20)	0 (0)	0 (0)
Hawaiian	1 (4.3)	0 (0)	0 (0)	0 (0)	0 (0)
Hispanic	4 (17.4)	1 (5.6)	1 (20)	0 (0)	2 (14.3)
Other	2 (8.7)	0 (0)	0 (0)	1 (9.1)	1 (7.1)
White	15 (65.2)	14 (77.8)	4 (80)	9 (81.8)	13 (92.9)

BPS, bladder pain syndrome; PP, pelvic pain without endometriosis or bladder symptoms; EAP, endometriosis-associated pain; EABP, endometriosis-associated pain and comorbid bladder symptoms; CON, free/bladder-symptom-free controls.

**Table 2:** Symptomatology across diagnostic groups. Data are presented as median (IQR) and range or as *n* (%) as appropriate.

	CON	Pain only		Pain + bladder	
		EAP	PP	EABP	BPS
EMLB	N/A	0 (0)	N/A	0 (0)	N/A
EMLU	N/A	6 (33.3)	N/A	5 (45.5)	N/A
BSS – symptoms (ICSI)*					
Median (IQR)	2 (2–4)	4 (1–5.5)	2 (1–2.5)	11 (9–12.5)	15 (11–17)
Range	0–7	0–6	1–3	9–13	10–18
Symptoms					
Urgency	0 (0)	1 (5.9)	0 (0)	5 (62.5)	7 (70)
Frequency	5 (22.7)	6 (35.3)	0 (0)	8 (100)	8 (80)
Nocturia	0 (0)	6 (35.3)	0 (0)	1 (14.3)	5 (50)
Dysuria	0 (0)	0 (0)	0 (0)	4 (50)	7 (70)
BSS – problem (ICPI)^†^					
Median (IQR)	0 (0–2)	1 (0–4)	0 (0–1)	9 (7–11.5)	13 (11.5–15)
Range	0–6	0–6	0–2	5–14	11–16
Problem					
Urgency	0 (0)	1 (5.9)	0 (0)	2 (25)	9 (90)
Frequency	0 (0)	2 (11.8)	0 (0)	2 (28.6)	9 (90)
Nocturia	0 (0)	1 (5.9)	0 (0)	4 (50)	7 (70)
Dysuria	0 (0)	0 (0)	0 (0)	6 (75)	9 (90)
GSRS					
Median (IQR)	1.28 (1.065–1.6675)	1.68 (1.4225–2.2775)	1.35 (1.1–1.835)	2.4 (1.725–3.95)	2.42 (1.33–3.07)
Range	1–3.17	1.07–4.25	1.05–2.22	1.1–4.48	1.23–5.57
Worst BPI^‡^					
Median (IQR)	4 (*n* = 1)	3.5 (2–5.5)	-	7 (4–8)	9 (7.5–9)
Range		2–6	-	3–9	6–9
Average BPI^‡^					
Median (IQR)	2 (*n* = 1)	1 (1–1.5)		5 (5–7)	7 (4–7)
Range		1–2		3–8	3–8
Bladder pain interference^§^					
Work or school	0 (0)	0 (0)	-	2 (22.2)	3 (42.9)
Daily activities at home	0 (0)	1 (6.7)	0 (0)	2 (18.2)	3 (42.9)
Sleep	0 (0)	1 (6.7)	0 (0)	4 (36.4)	3 (42.9)
Sexual intercourse	0 (0)	0 (0)	-	3 (33.3)	5 (71.4)
Exercise/sports	0 (0)	0 (0)	-	1 (11.1)	4 (57.1)
Social activities	0 (0)	0 (0)	-	1 (11.1)	3 (42.9)

EAP, endometriosis-associated pain; EABP, endometriosis-associated pain and comorbid bladder symptoms; EMLB, endometriosis lesions identified on bladder; EMLU, endometriosis lesions identified on the anterior cul-de-sac or ureteric lesions; BSS, bladder symptom score; BPI, bladder pain intensity; BPS, bladder pain syndrome; GSRS, gastrointestinal symptom rating score; PP, pelvic pain without endometriosis or bladder symptoms; CON, pain-free/bladder-symptom-free controls.

* ICSI total scores are given, followed by the *n* (%) of participants reporting ratings of 3, 4, or 5 to the symptom (about half the time/3 times per night/usually to almost always/5 or more times per night).

^†^ ICPI total scores are given, followed by the *n* (%) of participants reporting ratings of 3 or 4 (medium problem or big problem).

^‡^ Acute bladder pain intensity ratings over the last 3 months were assessed in those who reported experiencing pain in the bladder 3 or fewer months ago (i.e. not chronic bladder pain), using a numerical rating scale (0–10).

^§^ Bladder pain interference in the last 3 months was assessed in those who reported experiencing pain in the bladder 3 or fewer months ago, using a Likert scale (not applicable, not at all, slightly, moderately, quite a bit, and extremely). Shown is the number (%) reporting ‘quite a bit’ or ‘extremely’, showing high levels of interference; ‘–’ is given if missing response or no participant in the group reported pain in the bladder 3 months or fewer ago.

**Table 3:** Variables on the day of testing of the diagnostic groups. Data are presented as median (IQR) and range or as *n* (%) as appropriate.

	CON	Pain only		Pain + bladder	
		EAP	PP	EABP	BPS
Pain at time of testing NRS					
Median (IQR)	0 (0–0)	1 (0–3.5)	0 (0–1.5)	6 (1–7.5)	2 (0.5–6)
Range	0–1	0–5	0–2	1–8	0–9
State anxiety					
Median (IQR)	22 (20–31)	28 (25–30.5)	29 (22.5–33)	35 (26.5–41.5)	34 (20–48.5)
Range	20–35	23–34	21–35	22–45	20–55
Menstrual cycle phase					
Menstrual	3 (13.0)	0 (0)	0 (0)	0 (0)	0 (0)
Proliferative	3 (13.0)	0 (0)	0 (0)	2 (18.2)	0 (0)
Secretory	2 (8.7)	3 (16.7)	2 (40)	1 (9.1)	1 (7.7)
Unknown	2 (8.7)	0 (0)	0 (0)	0 (0)	2 (15.4)
Taking steroid hormones	12 (52.2)	15 (83.3)	3 (60)	8 (72.7)	10 (76.9)
Caffeine intake on day*					
0 cups	16 (69.6)	9 (50)	4 (80)	6 (54.5)	8 (57.1)
0–<2 cups	4 (17.4)	8 (44.4)	1 (20)	5 (45.5)	6 (42.9)
>2 cups	2 (8.7)	1 (5.6)	0 (0)	0 (0)	0 (0)

EAP, endometriosis-associated pain; EABP, endometriosis-associated pain and comorbid bladder symptoms; BPS, bladder pain syndrome; PP, pelvic pain without endometriosis or bladder symptoms; CON, pain-free/bladder-symptom-free controls.

* Number of cups of tea/coffee/equivalent.

### Comparing symptom groups: CON, ‘pain only’, and ‘pain + bladder’

There was a significant difference in age between the groups (*χ*^2^(2) = 9.25, *P* = 0.03), with the pain + bladder group (median: 37.5, IQR: 31.5–48) being significantly older than the pain only group (median: 33, IQR: 25.8–37) (*Z* = −2.46, *P* = 0.042) and the CON group (median: 30, IQR: 26–39) (*Z* = −2.74, *P* = 0.018). There was also a difference between the pain and pain + bladder group on parity although it did not withstand correction, with the pain + bladder group having greater parity (*Z* = −2.334, *P* = 0.060). Between these groups, there was also a difference in pain intensity rating on the day (*χ*^2^(2) = 23.3 *P* < 0.001), with significant differences between the CON group (median: 0, IQR: 0–0) and both the pain + bladder group (median: 2.5, IQR: 0–5.8) (*Z* = −4.56, *P* < 0.001) and the pain only group (median: 1, IQR: 0–2.3) (*Z* = −2.84, *P* = 0.012). There was a significant difference between the groups in state anxiety on the day of testing (*χ*^2^(2) = 8.28, *P* = 0.048), with post hoc tests showing a significant difference between the pain + bladder (median: 32, IQR: 20.3–41.8) and CON groups (median: 22, IQR: 20–31) (*Z* = −2.48, *P* = 0.039).

There were significant differences between the groups in the ICSI (*χ*^2^(2) = 31.55, *P* < 0.001; CON – median: 2, IQR: 0.5–4; pain only – median: 2, IQR: 1–5; pain + bladder – median: 12, IQR: 9.5–16), ICPI (*χ*^2^(2) = 32.58, *P* < 0.001; CON – median: 0, IQR: 0–2; pain only – median: 0, IQR: 0–2.8; pain + bladder – median: 12, IQR: 9–14.5), and GSRS (*χ*^2^(2) = 15.59, *P* < 0.001; CON – median: 1.3, IQR: 1.1–1.7; pain only – median: 1.6, IQR: 1.4–2.1; pain + bladder – median: 2.4, IQR: 1.7–3.1). Post hoc tests showed significant differences between the pain + bladder group and both pain only and CON groups for ICSI (*Z* = −4.89, *P* < 0.001, *Z* = −4.99, *P* < 0.001 respectively) and ICPI (*Z* = −4.83, *P* < 0.001, *Z* = −5.02, *P* < 0.001 respectively) and between pain + bladder and CON for GSRS (*Z* = −3.65, *P* < 0.001).

**Figure 1:**
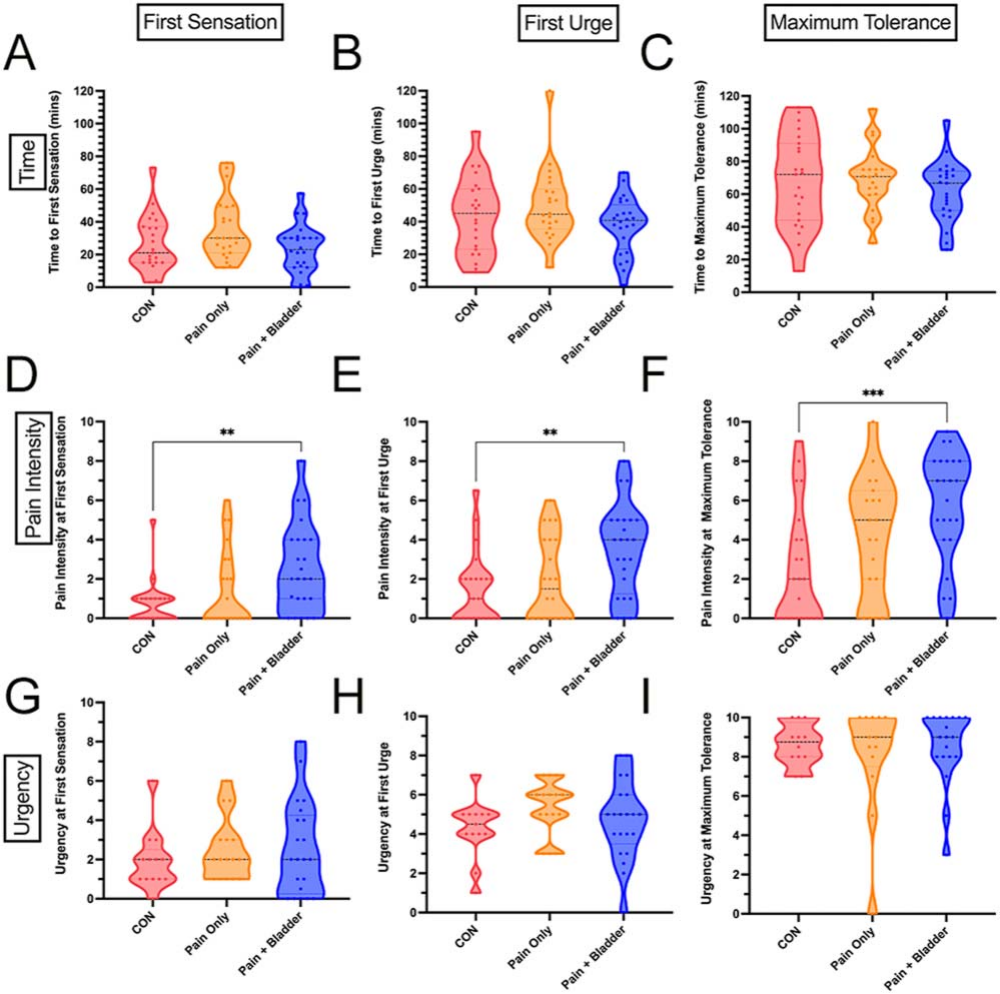
Time to reach each sensation is shown across the three symptom groups (A, B, C) as well as pain ratings (D, E, F) and urgency ratings (G, H, I) at each sensation. Pain intensity and urgency ratings were captured using a numerical rating scale (NRS) from 0 to 10. First sensation was described as ‘when riding in a car, the driver pulls over to a rest-stop to urinate, you would go as well’. First urge was described as ‘when riding in a car, you would initiate the request to find a rest-stop to urinate’. Maximum tolerance was described as ‘when riding in a car, you would urinate on the side of the road in bumper-to-bumper traffic’. For CON: *n* = 23; for pain only: *n* = 23; and for pain + bladder: *n* = 25. Not all participants reached maximum tolerance within the 2 h testing window (in the ‘pain only’: *n* = 3 and, in the ‘pain + bladder’: *n* = 2 did not reach maximum tolerance). Full breakdown of numbers of participants at each sensation can be seen in Supplementary Table 2. ‘Pain only’ are those with pelvic pain without bladder symptoms; ‘pain + bladder’ are those with pelvic pain and bladder pain, with urinary urgency and/or frequency symptoms; and CON are pain-free/bladder-symptom-free controls.

No statistically significant differences were found in the time taken to reach any of the sensations (FS, FU, and MT). Large variability was found within each group (Fig. 1A, B, C). However, significant differences were found between the groups in pain ratings for each sensation (Fig. 1D, E, F). The ‘pain + bladder’ group had significantly greater pain scores compared to CON at FS (*Z* = 3.12 *P* = 0.0054), FU (*Z* = 3.06 *P* = 0.0065), and MT (*Z* = 3.59 *P* = 0.001). The ‘pain only’ group were not significantly different to the other groups. We found no significant differences between the groups in urgency ratings at any sensation (Fig. 1G, H, I). Effect sizes for future sample size calculations for these can be found in Supplementary Table 4.

**Figure 2:**
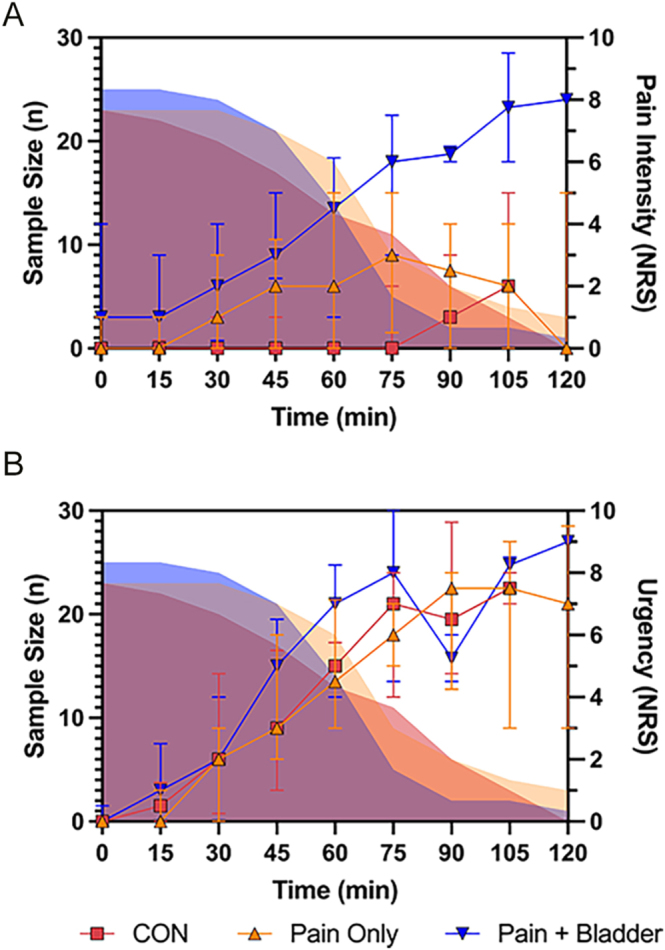
Number of participants giving ratings at each 15 min time point during the paradigm and pain intensity ratings (A) and urgency ratings (B). The number of participants (sample size, shown by coloured area) decreases over the course of the paradigm as participants reach maximum tolerance and thus end the paradigm. Shown as symbols is the median pain rating for the group, with error bars showing the IQR. The sample size is shown by filled in peaks. Pain intensity and urgency ratings were assessed using a numerical rating scale (NRS) score from 0 to 10. ‘Pain only’ are those with pelvic pain without bladder symptoms; ‘pain + bladder’ are those with pelvic pain and bladder pain, with urinary urgency and/or frequency symptoms; and CON are pain-free/bladder-symptom-free controls.

Pain intensity (A) and urgency (B) ratings throughout the paradigm in the symptom groups are shown in Fig. 2. We found that between the groups, there were no significant differences in the proportional-area-under-the-curve for urgency ratings (*χ*^2^(2) = 0.651, *P* = 0.722). However, for pain ratings, there was a significant difference between the groups (*χ*^2^(2) = 17.8, *P* = 0.0001), with a significant difference between the ‘pain + bladder’ and CON groups (*Z* = 4.20, *P* < 0.0001).

**Figure 3:**
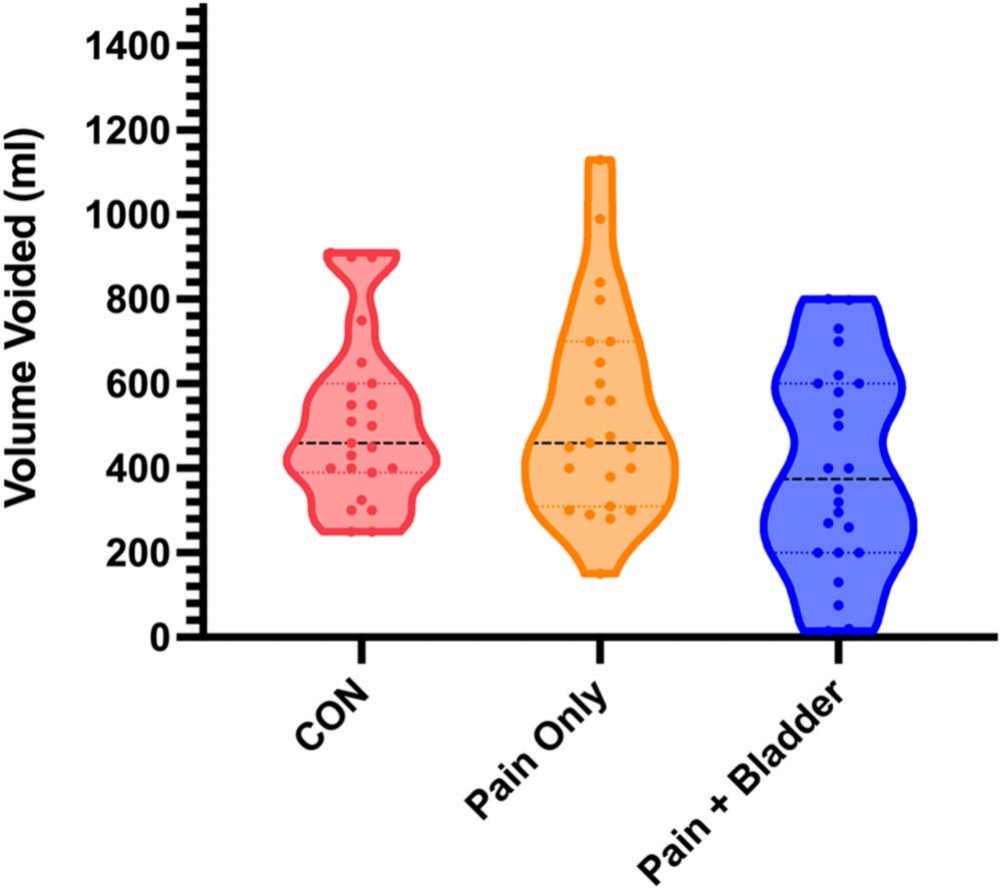
Volume voided at the end of paradigm between the three symptom groups. ‘Pain only’ are those with pelvic pain without bladder symptoms; ‘pain + bladder’ are those with pelvic pain and bladder pain, with urinary urgency and/or frequency symptoms; and CON are pain-free/bladder-symptom-free controls. For CON: *n* = 23; for pain only: *n* = 23; and for pain + bladder: *n* = 25.

We found no significant differences between the groups in the volume voided at the end, with large within-group variation (Fig. 3).

Given the significant difference in age across our groups, exploratory analysis was carried out exploring whether age correlated with any of time to sensation, pain and urgency ratings at sensations, and volume voided at the end of the paradigm (Supplementary Table 3); age was found to be significantly correlated with pain at FS but no other variables.

### Comparing diagnostic groups: EAP, EABP, BPS, and PP

**Figure 4:**
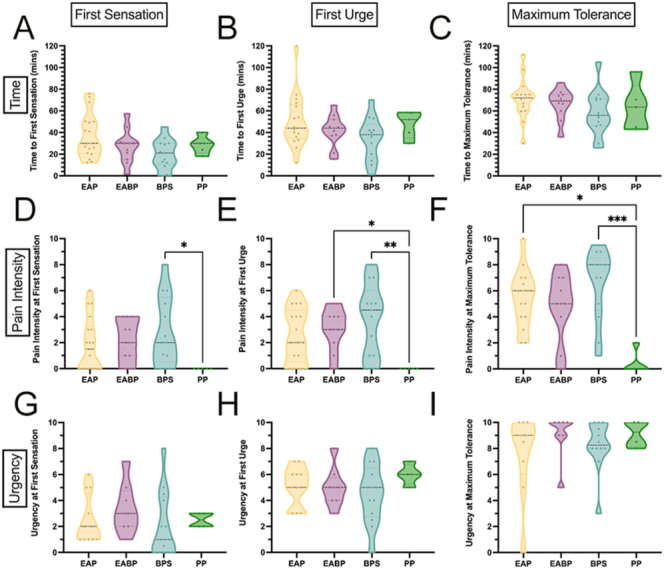
Across the four diagnostic groups are shown time to reach each sensation (A, B, C) as well as pain ratings (D, E, F) and urgency ratings (G, H, I) at each sensation. Pain intensity and urgency ratings were captured using a numerical rating scale (NRS) from 0 to 10. First sensation was described as ‘when riding in a car, the driver pulls over to a rest-stop to urinate, you would go as well’. First urge was described as ‘when riding in a car, you would initiate the request to find a rest-stop to urinate’. Maximum tolerance was described as ‘when riding in a car, you would urinate on the side of the road in bumper-to-bumper traffic’. For EAP: *n* = 18; for PP: *n* = 5; for EABP: *n* = 11; and for BPS: *n* = 14. Not all participants reached maximum tolerance within the 2 h testing window (in EAP: *n* = 3; in BPS: *n* = 1; and, in EABP: *n* = 1 did not reach maximum tolerance). EAP, endometriosis-associated pain; EABP, endometriosis-associated pain and comorbid bladder symptoms; BPS, bladder pain syndrome; PP, pelvic pain without endometriosis or bladder symptoms.

When subdividing the symptom groups to explore specifically whether including diagnosis of endometriosis as a stratifier impacted the results, we found no significant differences between the diagnostic groups and time to reach each sensation (Fig. 4).

The BPS group had significantly greater pain scores compared with the PP group at FS (*Z* = 2.76, *P* = 0.035), FU (*Z* = 3.26, *P* = 0.007), and MT (*Z* = 3.80, *P* = 0.0009). At FU, there was a significant difference between EABP and PP (*Z* = 2.68, *P* = 0.045). At MT, there was a significant difference between EAP and PP (*Z* = 2.85, *P* = 0.026). There were no significant differences between the diagnostic groups in urgency ratings at each sensation (*χ*^2^(3) = 3.89, *P* = 0.27 at FS; *χ*^2^(3) = 2.52, *P* = 0.47 at FU; *χ*^2^(3) = 2.42 *P* = 0.49 at MT).

**Figure 5:**
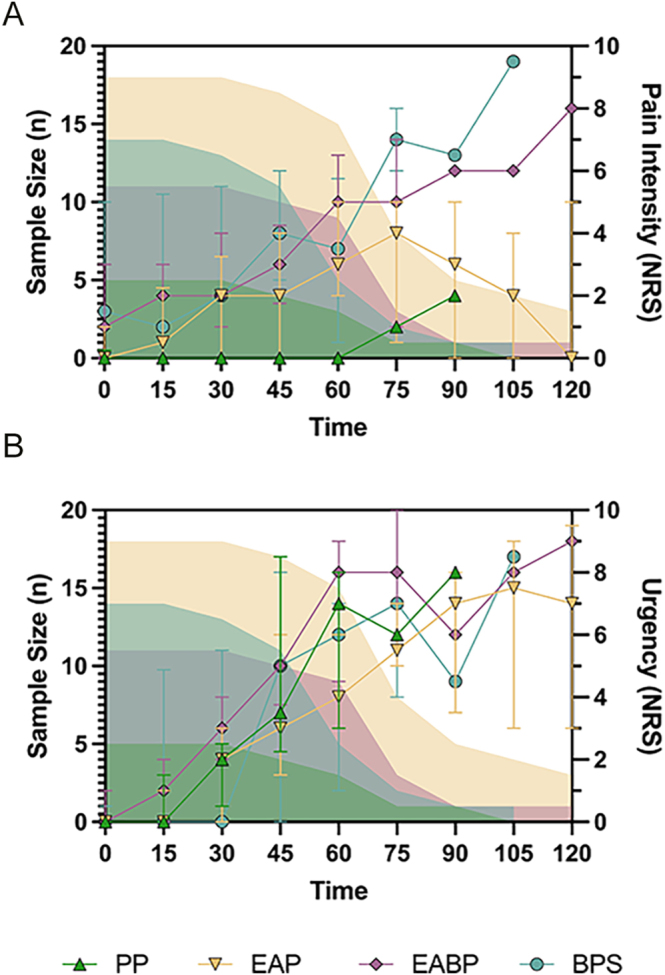
Number of participants giving ratings at each 15 min time point during the paradigm and pain intensity ratings (A) and urgency ratings (B). The number of participants (sample size) decreases over the course of the paradigm as participants reach maximum tolerance and thus end the paradigm. Shown as symbols is the median pain rating for the group, with error bars showing the IQR. The sample size is shown by filled in peaks. Pain intensity and urgency ratings were asked using a numerical rating scale (NRS) score from 0 to 10. EAP, endometriosis-associated pain; EABP, endometriosis-associated pain and comorbid bladder symptoms; BPS, bladder pain syndrome; PP, pelvic pain without endometriosis or bladder symptoms.

The pain and urgency ratings throughout the paradigm in the diagnostic groups are shown in Fig. 5. We found that between the diagnostic groups, there was no significant difference in the proportional-area-under-the-curve for urgency ratings (*χ*^2^(3) = 3.97, *P* = 0.27). However, a significant difference was found for the pain ratings (*χ*^2^(3) = 11.3, *P* = 0.010). Post hoc comparisons found the PP group to be significantly different to the EAP, BPS, and EABP groups (*Z* = −2.65, *P* = 0.042; *Z* = −2.73, *P* = 0.030; *Z* = −3.08, *P* = 0.0028, respectively).

**Figure 6:**
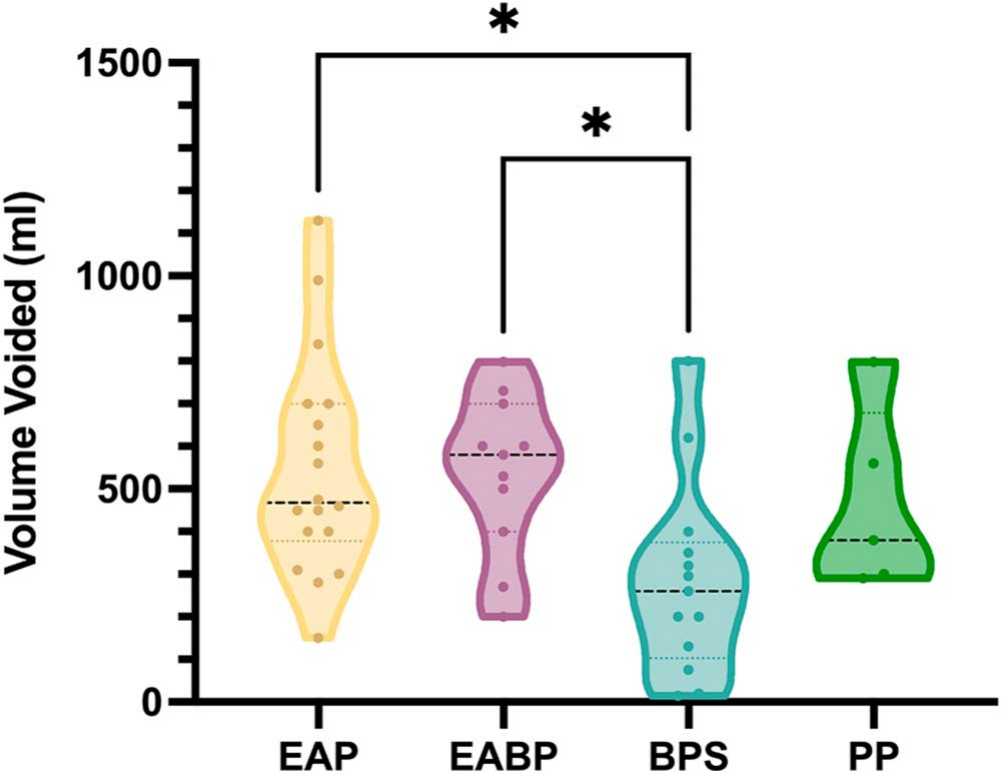
Volume voided at the end of the paradigm between the five diagnostic groups. *P < 0.05. For EAP: *n* = 18; for PP: *n* = 5; for EABP: *n* = 11; and for BPS: *n* = 14. EAP, endometriosis-associated pain; EABP, endometriosis-associated pain and comorbid bladder symptoms; BPS, bladder pain syndrome; PP, pelvic pain without endometriosis or bladder symptoms.

The BPS group had a significantly smaller volume voided compared to EAP and EABP (*Z* = 2.86, *P* = 0.026; *Z* = 2.65, *P* = 0.048, respectively) (Fig. 6).

### Correlation with questionnaire measures

In those with pelvic pain (i.e. excluding the CON group), there were significant correlations between paradigm pain ratings and the GSRS (gastrointestinal symptoms), but not the ICSI/ICPI (bladder symptoms) (after correction, these were between GSRS and pain ratings at FU and MT) (Table 4). There were no significant correlations with urgency ratings and any visceral symptom measures (Table 4).

**Table 4:** Spearman’s correlation coefficients (rho), corrected *P* values (*P*), and two-tailed 95% confidence intervals (CIs) for the correlation between pain and urgency ratings at each sensation and questionnaire measures, bladder symptom score (ICSI) and bladder bother score (ICPI) and gastrointestinal symptom rating scale (GSRS).

	rho	*P* (adjusted)	95% CI
Pain at first sensation			
ICSI	0.348	0.63	0.017–0.610
ICPI	0.385	0.306	0.065–0.634
GSRS	0.453	0.054	0.160–0.673
Urgency at first sensation			
ICSI	−0.320	>0.999	−0.631 to 0.080
ICPI	−0.243	>0.999	−0.573 to 0.154
GSRS	0.034	>0.999	−0.334 to 0.393
Pain at first urge			
ICSI	0.441	0.126	0.121–0.678
ICPI	0.460	0.072	0.150–0.687
GSRS	0.562	0.0029*	0.295–0.748
Urgency at first urge			
ICSI	−0.250	>0.999	−0.595 to 0.155
ICPI	−0.202	>0.999	−0.549 to 0.204
GSRS	−0.198	>0.999	−0.529 to 0.186
Pain at maximum tolerance			
ICSI	0.281	>0.999	−0.080 to 0.576
ICPI	0.435	0.18	0.103–0.679
GSRS	0.487	0.036*	0.184–0.705
Urgency at maximum tolerance			
ICSI	−0.312	>0.999	−0.656 to 0.139
ICPI	−0.204	>0.999	−0.577 to 0.239
GSRS	−0.327	>0.999	−0.641 to 0.081

* Adjusted P < 0.05.

In addition, there were no significant correlations between pain or urgency ratings at sensations and state anxiety score.

In exploratory correlations exploring the GSRS subscales (reflux, abdominal pain, indigestion, diarrhoea, and constipation) and pain/urgency ratings, we found significant (uncorrected) correlations between abdominal pain and pain ratings at FS (rho = 0.44, *P* = 0.004) and FU (rho = 0.39, *P* = 0.013); indigestion and pain ratings at FU (rho = 0.37, *P* = 0.016) and MT (rho = 0.35, *P* = 0.033); diarrhoea and urgency ratings at FS (rho = −0.36, *P* = 0.046), FU (rho = −0.53, *P* = 0.002), and MT (rho = −0.40, *P* = 0.037); pain ratings at FU (rho = 0.31, *P* = 0.049); constipation and pain ratings at FS (rho = 0.58, *P* < 0.001), FU (rho = 0.70, *P* < 0.001), and MT (rho = 0.51, *P* < 0.001); and urgency ratings at MT (rho = −0.43, *P* = 0.026).

## Discussion

### Main findings

Participants with bladder symptoms, regardless of endometriosis diagnosis, had greater pain scores throughout physiological bladder-filling. Interestingly, the volume voided (at MT or 2 h) was significantly different between BPS and EABP groups, despite them having the same symptom criteria and differing only in endometriosis diagnosis. This suggests that there may be different mechanisms underlying voiding behaviour and pain experience. Importantly, despite the presence of significant and impactful bladder symptoms in the EABP group, none had evidence of endometriosis lesions on their bladders. Correlations with symptom questionnaires suggest that this paradigm may be useful for exploring pelvic visceral sensitivity more broadly, rather than focussing only on the bladder.

### Results in the context of what is known

This study included a richly phenotyped and heterogeneous cohort of females with CPP, enabling us to explore the relationship between clinical measures and a non-invasive bladder sensitivity paradigm, allowing us to better understand its value.

The pain ratings during the paradigm related to clinical bladder symptomatology, which adds further face validity to this paradigm; we found significant differences in those who experience bladder symptoms (pain + bladder) and pain-free/bladder-symptom-free controls in pain ratings throughout. However, surprisingly, no differences were found in urgency ratings or time to reach sensations. This suggests, as seen in the work by the MAPP consortium (Griffith *et al.* 2016, Naliboff *et al.* 2017, 2022, Roy *et al.* 2021, Stephens-Shields *et al.* 2023), that there are different mechanisms driving pain and urgency symptoms in those with bladder symptoms. This supports the argument that urgency and pain symptoms should not be combined into a single outcome measure (Griffith *et al.* 2016), which is further reinforced by finding no correlations between measures in this paradigm and ICSI/ICPI scores, both of which combine urgency and pain components. We were surprised to see significant correlations between pain ratings and GSRS; however, viscero-visceral referral is common in CPP (Giamberardino *et al.* 2010) and, therefore, this paradigm may be a useful marker of generalised visceral sensitivity.

In previous work, Tu and colleagues have suggested that this paradigm can identify ‘silent’ bladder sensitivity, despite no reported bladder symptoms (Hellman *et al.* 2020). This may be similarly true of our EAP and PP groups, who did not report pain in the bladder nor urinary frequency/urgency, but all measures in this paradigm showed large variability within groups. Currently, it is not known whether those with ‘silent’ bladder symptoms have different mechanistic underpinnings to those with overt bladder symptoms, or whether those with ‘silent’ bladder symptoms may go on to develop overt symptoms.

Overall, these findings highlight the role of visceral sensitivity in CPP, particularly in those with bladder symptoms, which is consistent with other studies that have shown visceral sensitisation in IC/BPS (Grundy *et al.* 2018).

### Clinical implications

CPP conditions have historically been considered distinct pathologies with limited overlap in pain mechanisms. However, our findings, and others from the TRiPP consortium (Coxon *et al.* 2023, 2024*a*, Demetriou *et al.* 2023), highlight that whilst there are several pain mechanisms involved in CPP, these do not necessarily align with diagnostic groupings.

When subdividing based on endometriosis diagnosis, no significant differences were found in pain or urgency ratings during the paradigm; we did, however, find that the BPS group had a lower volume voided. This indicates different mechanisms driving pain on bladder filling and voiding behaviour. Importantly, a similar proportion of those with endometriosis with and without bladder symptoms had bladder/anterior cul-de-sac/ureteric lesions. These findings suggest that the endometriosis itself is not responsible for the burden of bladder symptoms seen in those with comorbid endometriosis and bladder symptoms.

### Research implications

The EABP group are particularly interesting in terms of the mechanisms giving rise to and maintaining pain. With the current work, it is not possible to determine whether they have a combination of mechanisms, which are associated with both endometriosis and IC/BPS, or whether there are distinct mechanisms giving rise to their phenotype. This is an area in which future research should be focussed, especially given this group seems to have a particularly high symptom burden (Demetriou *et al.* 2023), and the high rate of comorbidity between the two conditions (Wu *et al.* 2018, Namugosa *et al.* 2024).

Methods for stratification of individuals with chronic pelvic pain are currently at the forefront of research in this area, particularly in relation to treatment responses (Coxon *et al.* 2024*b*, Sandberg *et al.* 2025, Wolff *et al.* 2025). This paradigm is an example of one such method that may be useful in identifying whether individuals respond differently to standard treatment, based on their bladder sensitivity (Wolff *et al.* 2025). Within the framework of bladder pain that has been suggested (Wolff *et al.* 2025), particularly stratifying patients into bladder-centric and systemic symptoms, future work should determine whether this paradigm would be a helpful tool to categorise for treatment and, in addition, whether those with overt or ‘silent’ symptoms may benefit from treatments targeting bladder sensitivity (Lopez & Mangır 2021).

To explore the potential development of the described ‘silent’ symptoms, longitudinal research would be needed. In addition, larger cohorts would enable potential cut-offs to be determined to help identify such (sub) clinical phenotypes. Whilst currently, the paradigm is challenging to use for large data collection, due to the long timeframe (up to 2 h) needed with a trained individual collecting data throughout, it has the potential to be adapted to be done at home easily. This could improve its use for research, with much larger cohorts longitudinally and improved accessibility, and for clinical practice where it could be done by patients prior to visiting their healthcare provider.

Future research to understand the clinical applicability of this paradigm should determine whether responses to different treatments are predicted by measures collected during this paradigm and, thus, it could be used for personalising treatment regimes. In addition, it would be important to explore whether treatment of bladder sensitivity (even if silent) improves overall outcome for those with chronic pelvic pain and to investigate whether those with ‘silent’ symptoms are at an elevated risk of overt symptoms in the future.

### Strengths and limitations

This study, unlike validation studies of this paradigm (Tu *et al.* 2013), did not include three-dimensional ultrasound; thus, we were not able to assess the volume of the bladder during the paradigm and are limited to only final volume voided. This means that we cannot know the relationship between bladder volume and sensations, other than MT.

Whilst overall, our cohort was of reasonable size, particularly in comparison with studies using invasive visceral testing, when exploring differences between the 4 diagnostics groups, there are relatively small numbers in some groups. For many measures, we found large variation within groups, so further research in larger cohorts would be beneficial (Supplementary Table 4 has effect sizes for such calculations). In addition, we did not directly account for age and parity in our statistical tests.

Importantly, we did not perform a cystoscopy for all participants and therefore cannot know about the presence of Hunner’s ulcers or glomerulations in the bladder.

Measuring bladder sensitivity non-invasively is a known challenge, and therefore, this is an area of research that is particularly understudied in non-urological populations, such as those with endometriosis; thus, this study contributes to the field.

## Conclusion

This non-invasive bladder sensitivity paradigm can distinguish between those with both pain and bladder symptoms and pain-free/bladder-symptom-free controls. However, it may also be giving insight into different mechanisms or future trajectories for those with pelvic pain without overt bladder symptoms, whether or not this is associated with pathology. We found that those with pain and bladder symptoms were similar in pain ratings throughout the paradigm, regardless of whether they had an endometriosis diagnosis (including no relationship with lesions on the bladder itself and bladder symptoms). Ultimately, we hope that this information will allow us to better understand the underlying mechanisms of CPP and aid in the development of stratification methods.

## Supplementary materials



## Declaration of interest

LC reports research funding from UKRI outside of the submitted work. ET, MK, DP, LD, EE, CEL, QA, JB, LH, JM, JFG, AC, EP, PAM, and CBS report no competing interests. AH is an employee of Bayer AG, Germany. EPZ received financial support from Grunenthal and Mundipharma for research activities and advisory and lecture fees from Grünenthal, Novartis, and Mundipharma. In addition, she receives scientific support from the German Research Foundation (DFG), the Federal Ministry of Education and Research (BMBF), the German Federal Joint Committee (G-BA), and the Innovative Medicines Initiative (IMI) 2 Joint Undertaking under grant agreement No. 777500. This Joint Undertaking receives support from the European Union’s Horizon 2020 research and innovation programme and EFPIA. All money went to the institution EMP-Z is working for. RDT is in the advisory board for Bayer–IASP task force on chronic pain classification. AWH’s institution (University of Edinburgh) received payment for consultancy and grant funding from Roche Diagnostics to assist in the early development of a possible blood diagnostic biomarker for endometriosis. AWH’s institution (University of Edinburgh) received consultancy fees from Gesynta and Joii. AWH’s institution (University of Edinburgh) received grant funding from the UKRI, NIHR, CSO, and Wellbeing of Women for endometriosis research. AWH received payment for a lecture from Theramex and Gedeon Richter. AWH is an Editor-in-Chief of Reproduction and Fertility and was not involved in the review or editorial process for this paper, on which he is listed as an author. JV received research funding from Viatris and consultancy fees from AstraZeneca and Merz Therapeutics, outside of the submitted work. CMB received research grants from Bayer Healthcare, MDNA Life Sciences, Roche Diagnostics, European Commission, and NIH. His employer received consultancy fees from Myovant and ObsEva for work outside of this project. LAN received honoraria for consultancy and talks and associated travel expenses paid by Merz and Bayer. FC is a consultant and/or investigator for Allergan (Abbvie), Astellas, Bayer, Ipsen, and Recordati. SAM was an advisory board member for AbbVie and Roche and receives research funding from the National Institutes of Health, the US Department of Defense, the J Willard and Alice S Marriott Foundation, and AbbVie; none are related to the presented work. KTZ reports grant funding from EU Horizon 2020, NIH US, Wellbeing of Women, Bayer AG, Roche Diagnostics, Evotec-Lab282, and MDNA Life Sciences, outside the submitted work. JN is an employee of Merz Therapeutics GmbH, a shareholder of Bayer AG, Germany, and a shareholder of Eli Lilly. KV declares research funding from UKRI, NIHR, NIH US, and honoraria for consultancy and talks and associated travel expenses paid to her institution from Gedeon Richter, Gesynta, Reckitts, and Eli Lilly.

## Funding

This project received funding from the Innovative Medicines Initiative 2 Joint Undertaking under grant agreement No 777500. This Joint Undertaking receives support from the European Union’s Horizon 2020 research and innovation programme and EFPIA Companies. This project was pre-registered on clinicaltrials.gov: NCT04001244. Financial support was provided by the J Willard and Alice S Marriott Foundation for the establishment of and baseline data collection within the A2A cohort – from which the Boston-based TRiPP population was sampled. LC is currently funded by a grant from the Medical Research Foundation as part of the UKRI Strategic Priorities Fund (SPF) Advanced Pain Discovery Platform (APDP), a co-funded initiative by UKRI (MRC, BBSRC, and ESRC), Versus Arthritis, the Medical Research Foundation and Eli Lilly and Company Ltd (grant ref: MR/W02697/X1). Center for Neuroplasticity and Pain (CNAP) is supported by the Danish National Research Foundation (DNRF121).

## Author contribution statement

LC and KV conceived, planned, and carried out the study; analysed the data; and wrote the manuscript. ET, MK, and LD conceived and planned the study; analysed the data; and wrote the manuscript. PAM and JFG carried out the study and wrote the manuscript. CEL, DP, and AC planned and carried out the study and wrote the manuscript. JM, LAN, QA, CMB, JB, EE, AH, AWH, LH, SAM, EPZ, RDT, JV, KTZ, and JN conceived and planned the study and wrote the manuscript. EP planned the study; analysed the data; and wrote the manuscript. CBS and FC conceived, planned, and carried out the study and wrote the manuscript. All authors have given approval for the final version to be published and agree to be accountable for all aspects of the work in ensuring that questions related to the accuracy or integrity of any part of the work are appropriately investigated and resolved.
