# Supplementary material for: The value of a non-invasive bladder sensitivity paradigm in chronic pelvic pain
Source: Reprod Fertil. 2026 Mar 4;7(1):RAF250091. doi: 10.1530/RAF-25-0091 (PMC12974766; doi:10.1530/RAF-25-0091)
Supplement: Supplementary file 1 [file supplementary_materials.pdf]

**Pelvic Pain**
Please rate how severe your pelvic pain was at its worst during the last time you had vaginal intercourse/penetration using a scale from 0 to 10 where 0=no pain and 10=worst imaginable pain
Please rate how severe your pelvic pain was at its worst in the 24 hours after the last time you had vaginal intercourse/penetration using a scale from 0 to 10 where 0=no pain and 10=worst imaginable pain
Please rate how severe your pelvic pain with vaginal intercourse/penetration was when it was at its worst using a scale from 0 to 10 where 0=no pain and 10=worst imaginable pain
Please rate how severe your pelvic pain was at its worst in the last 3 months using a scale from 0 to 10 where 0=no pain and 10=worst imaginable pain
Please rate how severe your pelvic/lower abdominal pain was when it was at its worst using a scale from 0 to 10 where 0=no pain and 10=worst imaginable pain
If period in last 3 months and 'usually'/'always' experienced pelvic pain during period: Please rate how severe your pelvic pain was at its worst during your last period using a scale from 0 to 10 where 0=no pain and 10=worst imaginable pain
If period in last 3 months and 'usually'/'always' experienced pelvic pain during period: Please rate how severe your pelvic pain during your period was at its worst in the last 12 months using a scale from 0 to 10 where 0=no pain and 10=worst imaginable pain
If no period in last 3 months and 'usually'/'always' experienced pelvic pain during period when having them: Please rate how severe your pelvic pain during your period was when it was at its worst using a scale from 0 to 10 where 0=no pain and 10=worst imaginable pain
**Bladder pain**
Please rate how severe your worst bladder pain was in the last 7 days using a scale of 0 to 10.
**Urinary Symptoms**
In the past 7 days, how bothered were you by frequent urination during the daytime? 'Not at all'/'A little'/'Somewhat'/'Moderately'/'A great deal'
In the past 7 days, how bothered were you by having to get up during the night to urinate? 'Not at all'/'A little'/'Somewhat'/'Moderately'/'A great deal'
In the last 3 months, have you experienced difficulty passing urine? 'Yes'/'No'
In the last 3 months, have you experienced frequent bladder infections? 'Yes'/'No'
In the last 3 months, have you experienced still feeling full after urination? 'Yes'/'No'
In the last 3 months, have you experienced having to urinate again within minutes of urinating? 'Yes'/'No'

Supplementary Table 1: Symptom questions used to group participants into EAP, EABP PP, BPS and CON groups. In the CON group, they had to report pelvic and bladder pain <3/10 for all of the above questions, and report 'not at all'/'somewhat' or 'no' to all the urinary symptoms. The EAP and PP groups had to report at least one pelvic pain >4/10, with bladder pain <3/10 and no urinary symptoms. The EABP and BPS groups had to report at least one pelvic pain >4/10, bladder pain >4/10 and urinary symptoms ('Somewhat'/'Moderately'/'A great deal' to at least one, and 'yes' to at least one). Those in EAP and EABP had to in addition have a surgical diagnosis of endometriosis.

Variable	CON	Pain Only	Pain+Bladder
Time to FS	23	23	24
Pain at FS	23	23	24
Urgency at FS	13	16	21
Time to FU	23	22	24
Pain at FU	23	22	24
Urgency at FU	13	15	21
Time to MT	23	20	23
Pain at MT	23	20	23
Urgency at MT	12	13	19
Finished at 120mins	0	3	1
Volume at end	23	23	25

Supplementary Table 2: Number of participants giving ratings at each time point. Shown are the number of participants who reached each timepoint and whose data is included in analysis. FS = First Sensation, FU = First Urge, MT = Maximum Tolerance. ‘Pain Only’ are those with pelvic pain without bladder symptoms; ‘Pain+Bladder’ are those with pelvic pain and bladder pain, with urinary urgency and/or frequency symptoms; CON = pain-free/bladder-symptom-free controls.

Variable Correlated with Age	rho	p	n
Time to FS	0.056	>0.99	70
Pain at FS	0.380	0.01	70
Urgency at FS	0.073	>0.99	50
Time to FU	0.057	>0.99	69
Pain at FU	0.297	0.13	69
Urgency at FU	0.081	>0.99	49
Time to MT	0.066	>0.99	66
Pain at MT	0.287	0.19	66
Urgency at MT	-0.81	>0.99	44
Volume at end	-0.238	0.46	71

Supplementary Table 3: Correlation of variables with age across the cohort. FS = First Sensation, FU = First Urge, MT = Maximum Tolerance. Rho, adjusted p values and n shown.

	Kruskal-Wallis H	Degrees of freedom	p (unadjusted)	η^2
Pain at FS	10.60	2	0.005	0.154
Urgency at FS	0.87	2	0.648	0.018
Pain at FU	10.21	2	0.006	0.150
Urgency at FU	3.83	2	0.147	0.080
Pain at MT	13.02	2	0.001	0.200
Urgency at MT	0.218	2	0.897	0.005

Supplementary Table 4: Effect sizes of comparisons between CON, Pain and Pain+Bladder groups for future sample size calculations. FS = First Sensation, FU = First Urge, MT = Maximum Tolerance. Kruskal-Wallis H, degrees of freedom, unadjusted p values and eta squared (η^2) effect size shown.
